# S2Transformer: Exploring Sparsity in Remote Sensing Images for Efficient Super-Resolution

**DOI:** 10.3390/s25185643

**Published:** 2025-09-10

**Authors:** Zicheng Zhang, Hongke Xu, Shan Lin, Dejun Li, Yinghui Gao

**Affiliations:** 1 School of Electronics and Control Engineering, Chang’an University, Xi’an 710000, China; 2020032003@chd.edu.cn (Z.Z.); xuhongke@chd.edu.cn (H.X.); 2 Aviation University of Air Force, Nanhu Road Campus, Changchun 130022, China; 3 Academy of Military Science, Beijing 100080, China; yhgohp@yeah.net

**Keywords:** remote sensing, super-resolution, dynamic sparse transformer

## Abstract

Remote sensing image super-resolution (SR) techniques play a crucial role in geographic information analysis, environmental observation, and urban development planning. However, existing approaches are computationally intensive, which hinders them from bewing applied on resource-constrained devices. Although numerous efforts have focused on efficient image SR, the intrinsic sparsity characteristics of remote sensing images remain under-explored. To tackle these challenges, this paper introduces an efficient SR method founded on a dynamic Sparse Swin Transformer (S2Transformer). First, a dynamic sparse mask module is proposed to distinguish important regions from other ones. Subsequently, a dynamic sparse Transformer is developed to adaptively focus on important regions with more computational resources being allocated, markedly reducing redundant computations over background regions. Experiments are conducted on several benchmark remote sensing datasets and the results demonstrate that the proposed approach significantly outperforms existing methods in detail restoration, edge sharpness, and robustness, achieving superior PSNR and SSIM scores.

## 1. Introduction

Remote sensing images play a crucial role in various fields such as geographic information science, environmental monitoring, and urban planning. With the continuous advancement of remote sensing technology, the demand for high-resolution remote sensing images is increasing. However, due to the limitations of sensors, imaging conditions, and transmission bandwidth, the resolution of remote sensing images is often constrained, leading to the loss of image details. To address this issue, remote sensing image super-resolution (SR) has been widely applied to restore these missing details.

The research on remote sensing image SR can be dated back to the 1980s, initially focusing on traditional interpolation methods such as bicubic interpolation and nearest-neighbor interpolation. Motivated by the great success of deep learning, learning-based methods have been widely investigated and have gradually dominated the research of remote sensing image SR. Although multi-image SR demonstrates superior performance in the area of remote sensing image SR, acquiring multiple images is often challenging in the real world. Consequently, this paper focuses on single-image SR which has broad applicability in practical scenarios. Despite the promising results achieved by previous methods on benchmark datasets, the expensive computational cost of these methods hinders their application in resource-limited devices.

Recently, many efforts have been made to address this issue. Specifically, Shi et al. [1] proposed ESPCN, an efficient image SR network that accelerates the SR process by utilizing subpixel convolution. Motivated by the success of Transformers, Zhang et al. [2] proposed ELAN, which develops an efficient long-range attention module and acceleration mechanism to achieve efficient image SR. Later, Liu et al. [3] proposed a lightweight image SR method termed CATANet to efficiently aggregate content-similar tokens using a content-aware token aggregation (CATA) module. For remote sensing image SR, Peng et al. proposed CALSRN [4], which reduces the amount of parameters by about 30% while maintaining the quality of reconstruction by fusing the global features of the Swin Transformer with the local features produced by a CNN. Lin et al. proposed DTCNet [5], which distills knowledge from a Transformer-based teacher network to guide a lightweight CNN-based student network. Hou et al. proposed CSwT-SR [6], which combines spatial- and frequency-domain features in an amplitude-phase learning framework to achieve the enhancement of structural details.

**Figure 1:**
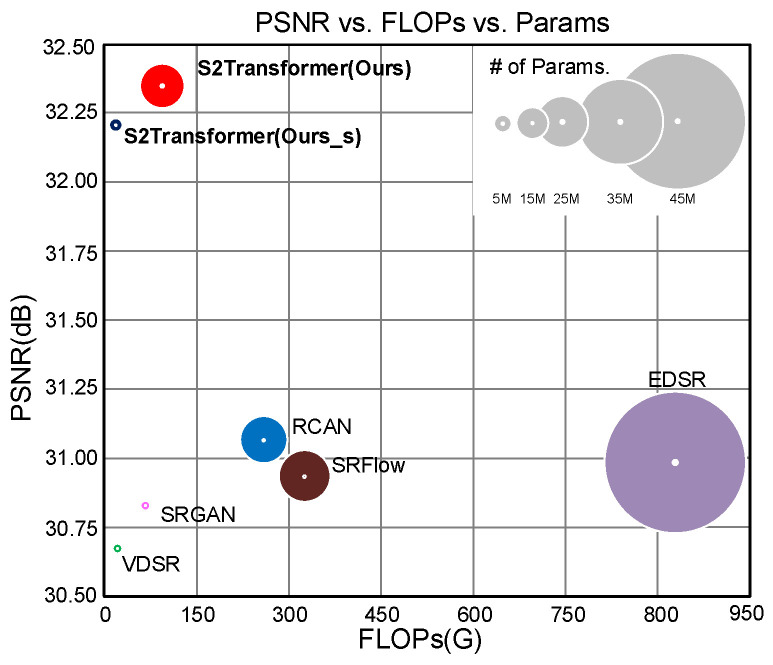
Quantitative comparison between our method and previous approaches. Our method well balances PSNR (dB) accuracy against parameter count and computational cost FLOPs (G), with metrics averaged across four remote sensing datasets.

Despite the progress made by the aforementioned methods, they commonly rely on techniques developed for general image SR and do not fully consider the characteristics of remote sensing images. In a remote sensing image, most regions are usually the background while targets of interests (e.g., airplanes, ships and cars) occupy only a few pixels. As a result, considerable redundant computational cost is involved in background regions. To remedy this, we introduce a Dynamic Swin Transformer that can dynamically allocate the computation on different regions according to their various importance. Particularly, we first develop a dynamic sparse mask module to produce a binary mask that distinguishes patches of higher importance from others in the image. Then, we propose a dynamic Swin Transformer block that can adaptively activate corresponding patches for subsequent computation based on the learned binary mask. In this way, redundant computation in background regions can be largely reduced to achieve significant speedup on edge devices without decreasing the accuracy of the reconstructed images. As shown in the Figure 1, our S2Transformer achieves the best balance between computational efficiency and reconstruction quality.

Overall, the contributions of this paper can be summarized as follows:

- We develop an efficient remote sensing image super-resolution network by exploiting the inherent sparsity of remote sensing images.
- We construct a dynamic sparse mask module to distinguish patches of high importance from others in an image, and then guide the Swin Transformer block to adaptively activate corresponding patches for efficient inference.
- We conduct extensive experiments on multiple remote sensing image datasets, validating the effectiveness and superiority of the proposed method.

## 2. Related Work

In this section, a brief review of existing image SR methods is first presented. Then, recent advances of diverse network acceleration techniques are discussed.

### 2.1. Image Super-Resolution

#### 2.1.1. General Image SR

Image super-resolution (SR) aims to reconstruct high-resolution (HR) images from their low-resolution (LR) counterparts. Over the last decade, significant advancements have been made in the field of general image super-resolution, with a shift from traditional techniques to deep learning approaches.

SRCNN [7] stands out as the pioneering CNN-based SR network, laying the foundation for learning-based methods. Later, VDSR [8] adopted a deeper network to achieve superior performance to SRCNN. Lim et al. [9] employed residual connections to build the developed EDSR with over 60 layers. Subsequently, Zhang et al. presented RCAN [10], which adopts a deep residual network structure with channel attention to focus on more informative features, which achieves much higher accuracy against previous approaches. Chen et al. proposed HAT [11] by combining channel attention with self-attention to better capture long-range correspondence in image SR tasks. SMSR [12] learns spatial masks to identify important regions and channel masks to prune redundant channels in unimportant regions. With the emergence of Transformers, many efforts have been made to introduce Transformers to image SR. Specifically, TTSR [13] was developed as the first Transformer-based SR model, which performs reference image super-resolution through rigid and soft attention modules. Inspired by the Swin Transformer, SwinIR [14] combined its local window self-attention with convolutional operations, and ESTNet [15] is an efficient Swin Transformer that uses an ECAB to select key channels and a group-wise multi-window self-attention (GAB) to strengthen cross-window modeling, achieving better RS image SR with lower computational cost, further enhancing SR performance. Recently, ESRT [16] optimized the structure by integrating CNN and Transformer features, reducing complexity.

With recent development of Mamba and Diffusion, Xia et al. proposed S3Mamba [17], which combines the scalable state-space model and a scale-aware self-attention mechanism to achieve high-quality arbitrary-scale SR with linear complexity. Di et al. developed QMambaBSR [18] that is capable of efficiently extracting subpixel information while mitigating the impact of noise interference. Liu et al. [19] introduced the first diffusion-based RSISR approach, which leverages low-resolution images as conditional inputs to produce high-resolution outputs.

#### 2.1.2. Remote Sensing Image SR

The success of learning-based image SR methods has promoted the research of remote sensing image SR (RSISR). Particularly, Leibel and Korner et al. [20] proposed the first CNN-based SR method for remote sensing images, termed msiSRCNN. Then, Xu et al. introduced DMCN [21], which performs feature integration by constructing local–global memory connections. Ren et al. [22] proposed ERCNN, which employs feature attention modules to optimize the mixed pixel problem. Dong et al. [23] proposed DSSR, which significantly improves the super-resolution of remotely sensed imagery through the introduction of a dense-sampling mechanism, a wide-feature attention module, and a chained training strategy. These methods have presented significant contributions in different remote sensing super-resolution tasks, especially in multi-scale feature extraction, residual learning and adaptive feature fusion, which significantly improve the quality and effectiveness of image reconstruction.

To leverage the great model capacity of Transformers, TransENet [24] was developed by employing a multi-stage enhancement architecture to integrate multi-scale features, overcoming the limitations of traditional models relying on upsampling layers and significantly improving RSISR performance. Subsequently, SLTN [25] constructed a multi-level feature extraction module guided by a spectral response function and employed Transformers for multi-layer nonlinear mapping learning. ESSAformer [26] combines a self-attention mechanism based on spectral correlation coefficients to improve computational efficiency and strengthen spectral feature interaction.

More recently, Mamba-based SR methods have also been investigated for remote sensing images. For instance, ConvMambaSR [27] integrates the state-space model (SSM) with a CNN to model global dependencies and extract local details, achieving better RSISR performance. Later, MambaFormerSR [28] further combines Mamba with a Transformer, designing a state-space attention fusion module and a convolutional Fourier feedforward network (CTFFN).

Although the aforementioned learning-based methods have achieved promising RSISR performance, these methods suffer form considerable redundant computation, which hinders their deployment on resource-limited devices. Despite the fact that several lightweight SR networks have been developed for general image SR, these methods do not fully consider the inherent characteristics of remote sensing images.

### 2.2. Network Acceleration

#### 2.2.1. Network Quantization

Network quantization techniques are primarily used to reduce storage cost and accelerate model inference, especially on resource-constrained devices. Quantization methods can be broadly divided into two categories: quantization-aware training (QAT) and post-training quantization (PTQ).

Quantization-aware training (QAT) introduces quantization operations during the training process, enabling the model to adapt to low-bit quantization while training. In the field of image SR, PAMS [29] proposes a tunable truncation parameter that dynamically adjusts the upper limit of the quantization range, thereby mitigating quantization errors. Then, DAQ [30] further employs channel-level distribution-aware quantization, which quantizes each channel based on its distribution characteristics. Subsequently, CADyQ [31] introduces a mix-bit quantization method that is able to dynamically allocate the bit widths to various regions according to their image contents.

Post-training quantization (PTQ) quantizes the model after training is completed, without the need to retrain the entire model. Instead, PTQ methods search for optimal quantization boundaries by minimizing the performance loss caused by quantization. As a pioneering PTQ method for image SR, DBDC+Pac [32] utilizes boundary compression and quantization calibration to reduce quantization loss. However, this method performs poorly on Transformer models, especially in handling activation values with long-tail distributions. Later, MinMax [33] employs a Min–Max quantization strategy, quantizing weights and activation values as integers within the minimum and maximum values.

#### 2.2.2. Network Pruning

Network pruning reduces computational and storage requirements by removing redundant neurons or connections in a neural network. Existing pruning methods are categorized into three groups: pruning before training (PBT), pruning during training (PDT), and pruning after training (PAT).

Pruning before training (PBT) refers to pruning before the training starts by selecting the least important connections to remove. Typically, SNIP [34] removes the least important weights by scoring once, avoiding the computational overhead during training. SynFlow [35] determines the pruning structure by evaluating the interactions between network layers. GraSP [36] stabilizes the training process by retaining the weights that contribute the least to the gradient signal.

Pruning during training (PDT) progressively removes unimportant connections during training. For example, SET [37] progressively prunes unimportant weights through dynamic sparse training while regenerating new weights during training. RigL [38] optimizes the pruning process by removing weights with the smallest gradients and gradually restoring sparse connections during training. Network slimming [39] introduces scaling factors for each channel and uses L1 regularization to selectively prune unimportant channels. MorphNet [40] forces network sparsity through the regularization of the batch normalization layers.

Pruning after training (PAT) occurs after training is completed, typically by removing redundant or unimportant weights to reduce the model size. Specifically, LTH [41] prunes the "winning subnet" and retrains it to recover original performance. FreeTickets [42] improves model performance by integrating multiple sparse sub-networks. Network pruning not only reduces the computational load but also speeds up inference, and it can decrease the model’s complexity without significantly affecting its accuracy.

#### 2.2.3. Sparse Inference

Sparse inference accelerates the inference efficiency of neural networks by skipping unnecessary computations. This technique exploits the abundance of zero elements in neural networks and reduces computational burden by optimizing the execution path. In recent years, sparse inference has been widely adopted to speed up neural networks. Willette et al. [43] proposed the Delta Attention method, which improves efficiency and accuracy in long-sequence inference by correcting the distribution shift of sparse attention while preserving high computational sparsity. Zhang et al. [44] proposed SpargeAtt, a sparse-quantized attention operator that requires no retraining. Gao et al. [45] proposed a lightweight and pluggable sparse-attention gating framework termed SeerAttention-R. Acharya et al. [46] proposed Star Attention, a two-stage block-sparse attention mechanism that combines “anchor-block” local encoding with distributed global querying.

## 3. Method

In this section, the overview of our proposed network is first introduced. Then, the structure details of our proposed dynamic sparse Transformer module and mask prediction block are presented.

### 3.1. Overview

**Figure 2:**
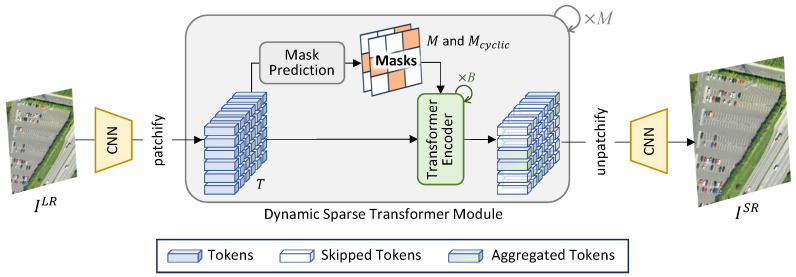
An overview of our proposed network. The input LR image is first fed to a CNN for feature extraction. Then, the resultant features are passed to *M* dynamic sparse Transformer modules and another CNN to reconstruct the SR result.

The overall framework of our method is illustrated in Figure 2. Given a low-resolution image $I^{LR}\in R^{H\times W\times 3}$, shallow features are first extracted through a convolutional neural network (CNN). Then, the resultant features are pathified to obtain tokens and passed to *M* dynamic sparse Transformer blocks (DSTMs), which is the core module of our network. Within each DSTM, the tokens are passed to the mask prediction module to produce a pair of binary masks ($M\in R^{\frac{H}{P}\times \frac{W}{P}}$ and $M^{shift}\in R^{\frac{H}{P}\times \frac{W}{P}}$, where *P* is the patch size), which are then incorporated by subsequent *B* Transformer blocks to achieve adaptive inference. Afterwards, the resultant tokens are unified and fed to another convolutional neural network (CNN) to generate the final high-resolution image $I^{SR}\in R^{sH\times sW\times 3}$.

### 3.2. Dynamic Sparse Transformer Module

**Figure 3:**
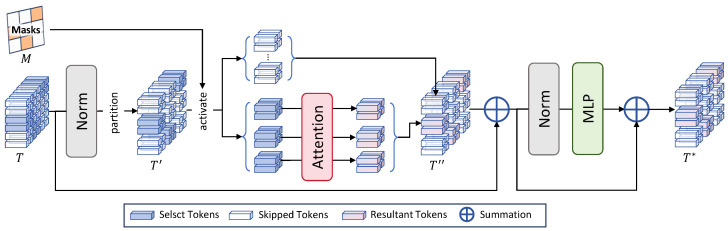
An illustration of our dynamic sparse transformer module.

As shown in Figure 2, the dynamic sparse transformer module is a core component of our network, which aims at activate important tokens for subsequent processing while keeping other ones untouched to improve computational efficiency. The detailed structure of this module is illustrated in Figure 3.

Fist, the input tokens $T\in R^{H\times W\times C}$ are fed to a layer normalization layer. Optionally, a cyclic shift is performed on the results following the Swin Transformer [47]. Then, the normalized tokens are partitioned into P×P patches, resulting in $R^{\frac{H}{P}\times \frac{W}{P}\times P\times P\times C}$. Afterwards, the corresponding binary mask $R^{\frac{H}{P}\times \frac{W}{P}}$ is employed to activate tokens in corresponding patches, which are then passed to an attention layer to capture the relationship between tokens in each patch. Meanwhile, other tokens skip the attention layer to recover the original shape of tokens $R^{\frac{H}{P}\times \frac{W}{P}\times P\times P\times C}$. In this way, only regions of higher importance are processed by the attention layer, striking a good balance between accuracy and efficiency. Afterwards, the results are passed through another layer normalization layer and Multilayer Perceptron (MLP), producing the final results.

#### Mask Predictor Block

**Figure 4:**
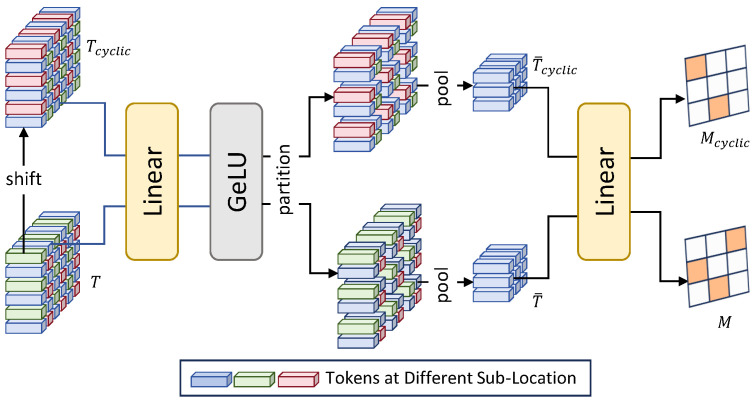
An illustration of our Mask Predictor Block.

Within each dynamic sparse Transformer module, a mask prediction block aims at identifying important patches in the input tokens to obtain binary masks. As shown in Figure 4, a cyclic shift is first performed on the input tokens. Next, both the original and shifted tokens are passed to a linear layer and a GeLU layer, which are then partitioned into patches of size $R^{\frac{H}{P}\times \frac{W}{P}\times P\times P\times C}$. Afterwards, average pooling is conducted on each patch to aggregate all tokens within the patch, producing $R^{\frac{H}{P}\times \frac{W}{P}\times C}$. Finally, the results are fed to the linear layer to generate a pair of binary masks.

Although binary masks are able to mark "important" patches out of other ones, they are inherently non-differentiable. To make the binary spatial mask learnable, a Gumbel softmax layer is employed. Specifically, the pooled tokens $R^{\frac{H}{P}\times \frac{W}{P}\times C}$ are fed to the linear layer to produce $R^{\frac{H}{P}\times \frac{W}{P}\times 2}$. Then, the Gumbel softmax trick is used to obtain a softened spatial mask $M\;\in \;R^{\frac{H}{P}\times \frac{W}{P}}$:

(1) $$M\left[x,y\right]=\frac{\exp\left(\left(F[x,y,1]\;+\;G[x,y,1]\right)/\tau \right)}{\sum_{i=1}^{2}\exp\left(\left(F^{spa}\left[x,y,i\right]\;+\;G\left[x,y,i\right]\right)/\tau \right)},$$

where x,y are vertical and horizontal indices, $G\;\in \;R^{\frac{H}{P}\times \frac{W}{P}}$ is a Gumbel noise tensor with all elements following a Gumbel(0,1) distribution and τ is a temperature parameter. When τ→∞, samples from the Gumbel softmax distribution become uniform. When τ→0, samples from the Gumbel softmax distribution become one-hot and binary masks can be obtained.

### 3.3. Loss Function

During the training phase, L1 loss between the SR result and the groundtruth is used for end-to-end optimization of the entire model. Compared to L2 loss, L1 loss avoids the blurring effect in our experiments.

## 4. Experiments

In this section, the implementation details are presented, including datasets, training settings, and evaluation metrics. Then, experiments are conducted to compare our proposed method against previous approaches. Finally, ablation experiments are conducted to study the effectiveness of our network designs.

### 4.1. Implementation Details

**Datasets:** In this section, our experiments are carried out on four widely-used remote sensing datasets: AID [48], DOTA V1.0 [49], DIOR [50], and NWPU-RESISC45 [51]. The specific details of each dataset are presented in Table 1.

- **AID**: This dataset contains 3000 training images and 900 test images, each at 600×600 pixels, with spatial resolutions ranging from 0.5 m to 8 m. It is designed for scene-classification tasks encompassing 30 categories.
- **DOTA V1.0**: This dataset contains 900 test images whose dimensions vary between 800 and 4000 pixels. This dataset targets object-detection tasks with 15 categories.
- **DIOR**: This dataset contains 1000 test images at 800×800 pixels. It is intended for object-detection tasks across 20 categories.
- **NWPU-RESISC45**: This dataset contains 315 test images at 256×256 pixels, with spatial resolutions from 0.2m to 30m. This dataset serves scene-classification tasks spanning 45 categories.

As illustrated in Table 1, these datasets span diverse spatial scales and task domains, thereby offering sufficiently heterogeneous data to evaluate the generality and effectiveness of the proposed method.

**Evaluation Metrics:** In this paper, the peak signal-to-noise ratio (PSNR) and structural similarity (SSIM) [52] are employed to evaluate the results. The PSNR is calculated as follows:
  (2) $$PSNR=20\times \log\left(\frac{MAX_{I}}{\sqrt{MSE}}\right),$$
  where MSE is the MSE between the SR result and the groundtruth, $MAX_{I}$ is the maximum pixel value. SSIM is computed as follows:
  (3) $$\mathrm{SSIM}\left(x,y\right)=\frac{\left(2u_{x}u_{y}+C_{1}\right)\left(2\sigma_{xy}+C_{2}\right)}{\left(u_{x}^{2}-u_{y}^{2}+C_{1}\right)\left(\sigma_{x}^{2}-\sigma_{y}^{2}+C_{2}\right)},$$
  where $u_{x}$ and $u_{y}$ are the pixel means of images *x* and *y*, $\sigma_{xy}$ is the covariance of images *x* and *y*, $\sigma_{x}$ and $\sigma_{y}$ are the variances corresponding to images *x* and *y*, and C1 and C2 are non-zero constants.
**Model Details:** In our experiments, the window size of our model is set to 8 and the MLP expansion ratio is fixed at 4. For our S2Transformer (Ours), the embed dimension is set to 180, the number of Transformer layers is set to 6 and the number of attention heads is set to 4. For the lightweight S2Transformer (Ours_s), the embed dimension is set to 60, the number of Transformer layers is set to 4 and the number of attention heads is set to 6.
**Training Details:** Our experiments were performed on two NVIDIA GeForce RTX 4090 GPUs (Santa Clara, CA, USA). During the training phase, the AdamW optimizer was adopted to train the model with batch size set to 24. The learning rate was initialized as $2\times 10^{-4}$. The training was stopped after 500 epochs.

### 4.2. Performance Evaluation

#### 4.2.1. Bicubic Degradation

We first compare our S2Transformer with previous state-of-the-art SR methods on widely applied bicubic degradation. Table 2 summarizes the SR performance of different methods achieved on 30 distinct scene categories of the AID dataset. As we can see, compared with the HAT, this study attains the highest PSNR and SSIM scores across different scene categories and clearly outperforms EDSR, RCAN, HAT, and other baselines with notable margins. It achieves the largest PSNR gains in structure-intensive scenes such as Airport (+0.98 dB), Parking (+0.83 dB) and D-Residential (+0.92 dB), indicating that extra computation is indeed routed to fine man-made details. Improvements are also consistent—though smaller—in low-texture classes like Desert (+0.52 dB) and Meadow (+1.26 dB), showing that the model avoids over-processing homogeneous backgrounds while still preserving radiometric fidelity.

**Figure 5:**
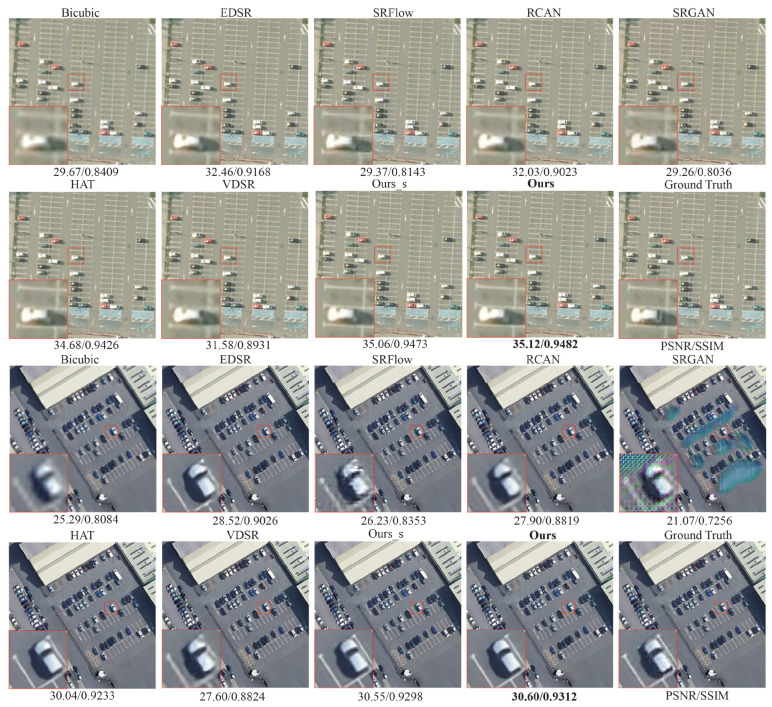
Visualization ×4 results using different methods on “parking_251” and “parking_314” samples of AID. The best result PSNR/SSIM is shown in **boldface**. Magnify to get a clearer view.

We further visualize the results produced by different methods in Figure 5. It can be observed that bicubic interpolation produces blurring artifacts, yielding poor visual quality and correspondingly low PSNR and SSIM scores. EDSR and RCAN can recover some details, yet they still exhibit noticeable blurring at the boundaries of intricate textures or fine objects. Although SRFlow and SRGAN provide a certain degree of visual improvement, their overall PSNR/SSIM metrics remain sub-optimal. By contrast, the our proposed method more effectively restores fine details and produces sharper, clearer edges, yielding visuals that closely approximate the ground truth and achieving the highest PSNR and SSIM values. For example, in the first image set, Ours attains 35.12 dB/0.9482, clearly surpassing HAT (34.68 dB/0.9426) and RCAN (32.03 dB/0.9023). These results clearly demonstrate the stability and robustness of our method for remote sensing image SR.

Table 3 presents the performance of various methods on the AID, DOTA, DIOR, and NWPU-RESISC45 datasets. Our proposed method attains the best overall performance with a moderate parameter count of 16.01 million. Meanwhile, the lightweight version of Ours_s contains merely 4.16 million parameters, yet still delivers highly competitive results. Averaged over all datasets, Ours clearly surpasses widely used CNN/Transformer baselines (e.g., HAT: 31.22/0.8201, RCAN: 31.13/0.8160, SwinIR: 31.16/0.8188, ESRT: 31.17/0.8189, ESTNet: 31.55/0.8263, SMSR: 31.59/0.8272). The lightweight version of Ours_s further reduces computation to 8.73G FLOPs yet remains in the leading cluster, demonstrating the method’s efficiency and suitability for resource-constrained scenarios.

**Figure 6:**
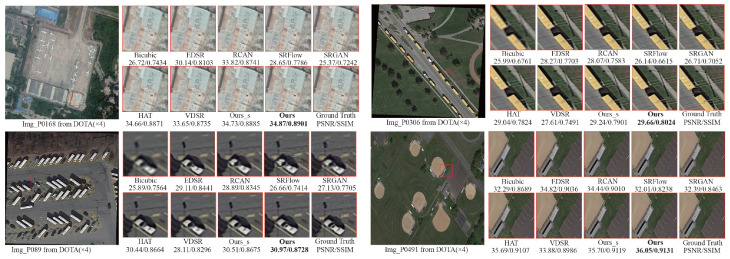
Visualization ×4 results using different methods on DOTA V1.0. The best PSNR/SSIM is shown in **boldface**. Magnify to get a clearer view.

Figure 6 illustrates the visual results and corresponding quantitative metrics (PSNR/SSIM) achieved on the DOTA V1.0 dataset. Qualitatively, bicubic interpolation yields pronounced blurring and detail loss especially along object boundaries and within texture-rich areas resulting in inferior visual quality. Methods such as SRFlow and SRGAN offer some visual improvement, yet they still suffer from inadequate detail restoration, for instance, boundary blurring and shape distortion of targets with SRGAN exhibiting marked instability in certain images. EDSR and RCAN perform relatively better in edge sharpening and detail recovery, but still fall short of the desired level of finesse. In contrast, Ours surpasses all competitors in both visual fidelity and quantitative metrics. For example, on “Img_P0168”, Ours attains 34.87 dB/0.8901, outperforming HAT (34.66 dB/0.8871) and RCAN (33.82 dB/0.8741) and delivering crisper object boundaries and richer texture details. Moreover, lightweight Ours_s also demonstrates strong performance even under constrained computational settings, highlighting the efficiency and practicality of our approach.

**Figure 7:**
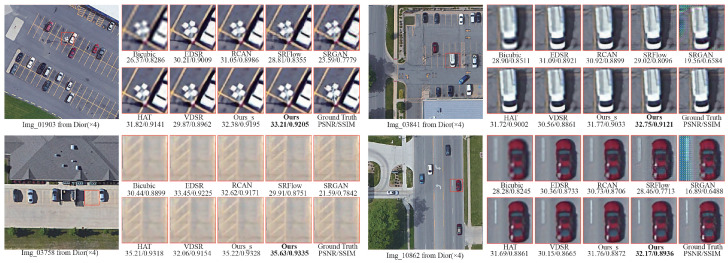
Visualization ×4 results using different methods on DIOR. The best PSNR/SSIM is shown in **boldface**. Magnify to get a clearer view.

It is evident in Figure 7 that bicubic interpolation suffers from pronounced blurring and detail loss on the DIOR dataset, yielding the poorest visual quality. EDSR and RCAN recover certain details effectively, yet they remain deficient in restoring fine textures and crisp edges; for instance, in Img_01903 and Img_03841, target boundaries still exhibit blurring or artifacts. However, SRFlow and SRGAN produce noticeable texture distortions and artifacts across multiple images (e.g., Img_03841 and Img_10862), resulting in substantially lower PSNR and SSIM scores. By contrast, Ours exhibits clear superiority in both visual fidelity and quantitative performance. For example, on Img_03758, Ours achieves the highest score of 35.63 dB/0.9335, surpassing the next-best HAT (35.21 dB/0.9318) and RCAN (32.62 dB/0.9171).

**Figure 8:**
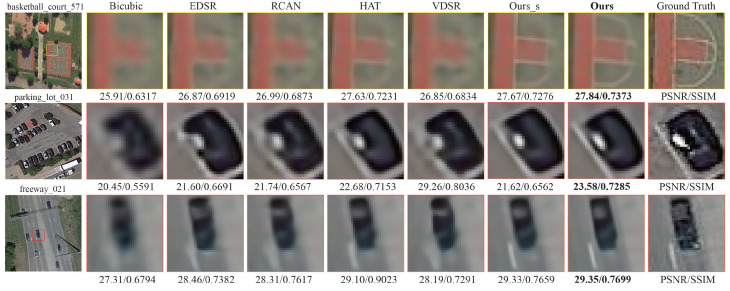
Visual x4 comparisons on NWPU-RESISC45.

We also visualize the results achieved on the NWPU-RESISC45 dataset in Figure 8. Bicubic interpolation yields the lowest image quality, with substantial blurring of details particularly along object boundaries and within textured areas, as is evident in the “basketball_court” and “freeway” scenes. EDSR, RCAN, and VDSR improve sharpness to some extent, yet they remain deficient in texture fidelity and contour clarity, performing poorly in complex scenarios such as “parking_lot.” By contrast, Ours yields pronounced visual improvements; for instance, in “basketball_court,” it achieves the highest score of 29.35 dB/0.7699. Even in the relatively complex “freeway” scene, Ours attains the top score of 27.84 dB/0.7373, attesting to its robustness and generalization capability. Moreover, the lightweight Ours_s exhibits consistently strong performance, underscoring the efficiency of the proposed dynamic sparse Transformer architecture.

#### 4.2.2. Realistic Degradations

Following [53], experiments are also conducted on realistic degradations. Specifically, HR images are first blurred using 21×21 Gaussian kernels and then bicubicly downsampled with noises being added. The width of the Gaussian kernel is determined by a Gaussian probability density function that follows a normal distribution N(0,Σ). Here, Σ is the covariance, which is determined by two random eigenvalues $\lambda_{1},\lambda_{2}∼U\left(0.2,4\right)$ and a random rotation angle θ∼U(0.π). The noise level ranges within [0, 25].

Table 4 presents the results achieved on the DIOR dataset under varying noise levels (0,5,10) and Gaussian blur kernels. Ours exhibits the highest robustness across all noise levels and anisotropic blur settings, with PSNR values that substantially surpass those of competing approaches such as EDSR, RCAN, VDSR, and HAT. For instance, at a noise level of 0, Ours attains an average PSNR of 31.21dB, whereas the runner up reaches only 31.00 dB. This margin widens at higher noise levels (5 and 10), further underscoring the superiority of the proposed method.

**Figure 9:**
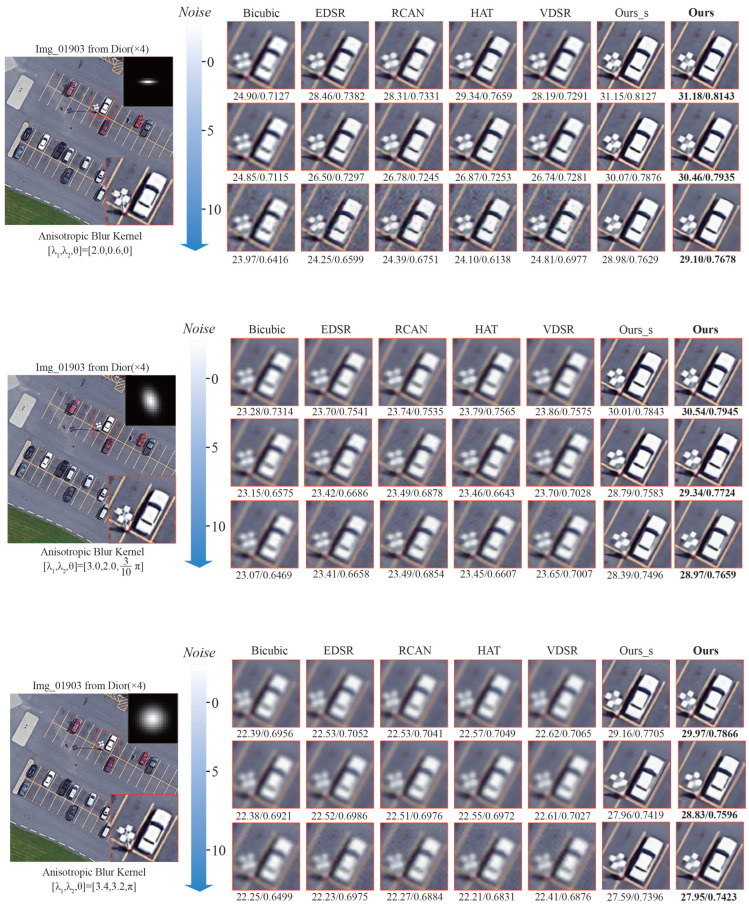
The visual comparison of experiments on noises and anisotropic Gaussian blur. This image, “Img_01903”, is taken from the DIOR dataset. The best PSNR/SSIM is shown in **boldface**.

We further visualize the results in Figure 9. It can be observed that Figure 9 illustrates the visual restoration results and quantitative metrics (PSNR/SSIM) of different methods. As noise and blur intensities increase, previous methods experience a marked decline in their ability to restore fine details. For instance, at a noise level of 10, images reconstructed by these methods exhibit pronounced blurring and structural loss, with PSNR and SSIM scores dropping appreciably. By contrast, our proposed method demonstrates pronounced robustness across all degradation settings and effectively restores image details while sustaining high visual fidelity and metric scores. Concretely, under the first anisotropic Gaussian kernel ([ $\lambda_{1},\lambda_{2},\theta$ ] = [2.0,0.6,0]), Ours attains 31.18 dB/0.8143, outperforming the runner-up Ours_s (31.15 dB/0.8127) and all other methods. This advantage persists at a noise level of 10, underscoring our proposed method’s strong noise-resilience.

**Figure 10:**
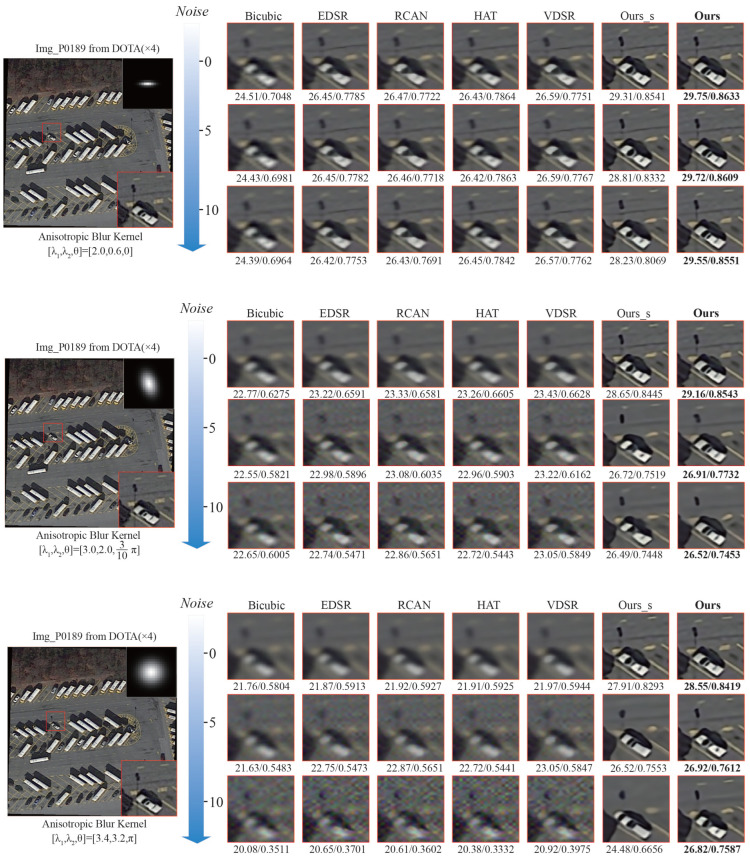
The visual comparison of experiments on noises and anisotropic Gaussian blur. This image, “Img_P0189”, is taken from the DOTA V1.0 dataset. The best PSNR/SSIM is shown in **boldface**.

We also further visualize the results achieved by different methods in Figure 10. It can be observed that, in the presence of increasing noise, the performance of all models decreases, and Ours decreases the least, showing greater robustness. Under the same noise and blur conditions, Ours consistently achieves the highest PSNR and SSIM values, followed by Ours_s. For example, under the first anisotropic Gaussian kernel ([ $\lambda_{1},\lambda_{2},\theta$ ] = [3.4,3.2,π ]) in extreme conditions of high noise (Noise = 10) and strong blurring, the advantages of Ours are even more pronounced. These results further validate the effectiveness and superiority of our method.

In summary, the visual and quantitative results consistently highlight the superiority of Ours across diverse noise and blur conditions, further confirming the broad applicability of our model to complex remote sensing image degradations.

### 4.3. Model Analyses

#### 4.3.1. Mask Predictor Block

To verify the effectiveness of the Mask Predictor Block (MPB), we conducted an ablation study in Table 5. When the MPB is removed (Baseline), the model processes all regions equally and suffers a huge computational cost. Once the MPB is introduced, Ours maintains competitive performance with FLOPs being significantly reduced. This is because that MPB can well recognize the importance of different regions and can focus on those with high object existence probability. These findings conclusively demonstrate the effectiveness of our MPB.

#### 4.3.2. Visualization of Learned Masks

**Figure 11:**
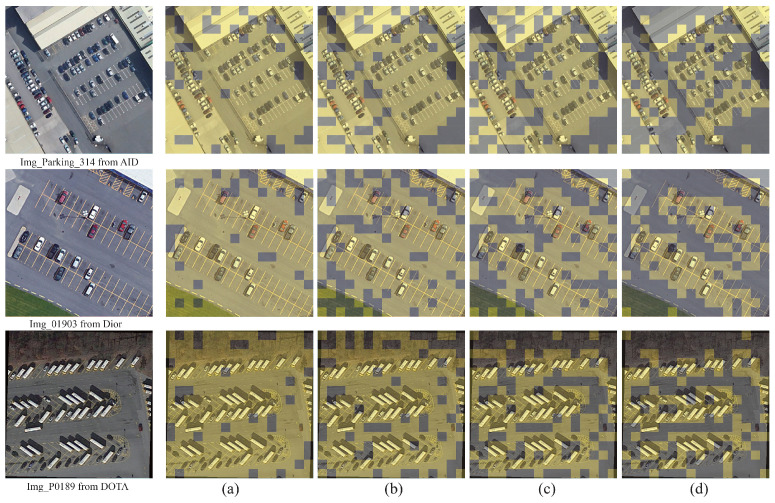
Visualization of learned sparse masks. (**a**–**d**) visualize the masks produced at stages 1–4.

We further visualize our learned masks in Figure 11. As we can see, our mask prediction module is able to identify patches with texture-rich edges and targets very well. For example, on Img_Parking_314, we can see that vehicles in the parking lot are recognized as important regions. Furthermore, as the network depth increases, the learned mask gradually focuses on fewer regions. By skipping the computation of these patches, inference efficiency can be improved while maintaining superior performance.

## 5. Discussion

Our approach differs from prior RSISR methods by introducing a dynamic, block-wise sparse inference mechanism. Conventional baselines (e.g., SwinIR [14]) execute dense self-attention over all regions without considering their different image contents. To improve the efficiency, many efforts rely on network pruning [54,55,56], network quantization [29,57,58] or lightweight designs [59,60]. For example, ESTNet [15] develops group-wise attention and channel attention modules to boost the efficiency for Transformer-based SR. A related prior work on SMSR [12] reveals that most LR images contain large flat regions where dense computation is wasteful. However, SMSR is specially developed for CNN-based structures and cannot be directly extended to Transformers. In this paper, S2Transformer learns binary, patch-level spatial masks that route only high-importance regions through attention and MLP branches, while bypassing low-importance areas with a light path. This content-adaptive approach aligns better with the sparse nature of remote sensing imagery, which typically contains large homogeneous areas (e.g., sea, cropland, desert) with sparse but critical objects (e.g., roads, buildings, vessels).

Compared with the baseline SwinIR, the key difference is a shift in the inference paradigm rather than the choice of backbone. SwinIR centers on local window self-attention with shifted windows and convolutions but treats all tokens uniformly, making computation weakly coupled to content. In contrast, our model explicitly predicts binary masks per block to preserve tha coverage of cross-window salient regions under the shifted-window scheme. Then, only activated tokens are processed by attention, while non-activated tokens follow a lightweight recovery path. As a result, our method maintains a high reconstruction quality while substantially reducing its redundant computation and memory footprint, offering a more practical efficiency–accuracy trade-off for resource-constrained deployments.

## 6. Conclusions

This paper proposes an efficient remote sensing image SR approach founded on a dynamic Sparse Swin Transformer (S2Transformer), which is tailored to exploit the sparsity of target regions and the redundancy of background information in remote sensing images. Our sparse Transformer blocks can adaptively identify regions of interest and markedly reduces the computational load on background areas, thereby boosting efficiency while preserving high-quality reconstructions. Experiments on remote sensing datasets show that the proposed approach delivers marked gains in visual quality, quantitative metrics, and robustness. Additional studies under various noise levels and blur degradations confirm that the method reliably recovers fine details even in complex scenarios.

## Figures and Tables

**Table 1:** A detailed summary of the dataset attributes, including AID, DOTA, DIOR, and NWPU-RESISC45.

	Train	Test			
Dataset	AID [48]	AID [48]	DOTA V1.0 [49]	DIOR [50]	NWPU-RESISC45 [51]
Used (Total) Images	3000 (10,000)	900 (10,000)	900 (2806)	1000 (23,463)	315 (31,500)
Image Size	600×600	600×600	800∼4000	800×800	256×256
Resolution	0.5∼8m	0.5∼8m	-	-	0.2∼30m
Categories	30	30	15	20	45
Task	Scene Classification	Scene Classification	Object Detection	Object Detection	Scene Classification

**Table 2:** Quantitative results on the AID test set. Here we report the PSNR/SSIM performance of SISR models on 30 classes of scenes. The best result is shown in **boldface**.

Land Cover	Bicubic		EDSR		VDSR		SRFlow		RCAN		SRGAN		HAT		This Study	
	**PSNR**	**SSIM**	**PSNR**	**SSIM**	**PSNR**	**SSIM**	**PSNR**	**SSIM**	**PSNR**	**SSIM**	**PSNR**	**SSIM**	**PSNR**	**SSIM**	**PSNR**	**SSIM**
Airport	27.83	0.7554	29.93	0.8282	30.12	0.8301	28.97	0.7916	30.13	0.8318	30.29	0.8416	30.15	0.8319	**31.13**	**0.8571**
Bare Land	35.60	0.8564	36.94	0.8837	36.18	0.8854	35.75	0.8634	36.99	0.8844	35.86	0.8624	36.88	0.8841	**37.39**	**0.8987**
Baseball Field	31.00	0.8305	33.05	0.8765	32.16	0.8351	32.89	0.8687	33.30	0.8789	32.78	0.8635	33.25	0.8789	**33.81**	**0.8817**
Beach	32.90	0.8446	34.18	0.8727	34.19	0.8754	34.13	0.8706	34.33	0.8751	34.26	0.8814	34.34	0.8756	**34.99**	**0.8849**
Bridge	30.22	0.8283	32.93	0.8800	32.91	0.8795	32.57	0.8653	33.13	0.8819	32.64	0.8689	33.04	0.8809	**33.68**	**0.8946**
Center	26.51	0.6944	28.77	0.7921	28.68	0.7859	27.65	0.7721	28.96	0.7966	27.47	0.7671	28.92	0.7956	**29.67**	**0.8159**
Church	24.29	0.6333	26.30	0.7469	26.35	0.7519	25.91	0.7389	26.54	0.7529	25.93	0.7409	26.56	0.7532	**27.75**	**0.7758**
Commercial	27.33	0.7174	29.01	0.7940	28.98	0.7915	28.96	0.7840	29.24	0.8000	29.36	0.7978	29.21	0.8007	**30.29**	**0.8179**
D-Residential	22.93	0.5671	24.38	0.6839	24.71	0.6926	24.15	0.6737	24.63	0.6930	24.37	0.6889	24.67	0.6936	**25.59**	**0.7316**
Desert	39.26	0.9100	40.20	0.9268	40.05	0.9175	40.03	0.9218	40.24	0.9272	40.56	0.9356	40.37	0.9278	**40.89**	**0.9413**
Farmland	33.10	0.8226	35.00	0.8683	34.81	0.8628	34.86	0.8616	35.11	0.8701	34.06	0.8349	35.03	0.8691	**35.68**	**0.8824**
Forest	28.79	0.6605	29.85	0.7315	29.76	0.7286	29.45	0.7115	29.95	0.7345	29.15	0.7019	30.01	0.7363	**30.49**	**0.7595**
Industrial	26.77	0.6952	28.88	0.7931	28.67	0.7927	28.68	0.7731	29.04	0.7977	28.61	0.7721	29.04	0.7980	**29.98**	**0.8159**
Meadow	33.86	0.7483	34.63	0.7804	34.59	0.7795	34.37	0.7704	34.65	0.7815	34.29	0.7694	34.70	0.7815	**35.96**	**0.8023**
M-Residential	26.36	0.6335	28.34	0.7365	28.31	0.7349	28.17	0.7267	28.52	0.7415	28.08	0.7159	28.46	0.7408	**29.65**	**0.7610**
Mountain	29.51	0.7349	30.63	0.7885	30.60	0.7849	30.26	0.7785	30.72	0.7908	30.31	0.7816	30.78	0.7923	**31.59**	**0.8098**
Park	29.06	0.7530	30.54	0.8130	30.51	0.8009	30.01	0.7954	30.72	0.8170	30.09	0.8004	30.71	0.8189	**31.56**	**0.8327**
Parking	24.24	0.7060	27.25	0.8317	27.19	0.8294	27.11	0.8287	27.50	0.8372	27.16	0.8297	27.56	0.8405	**28.39**	**0.8585**
Playground	32.64	0.8450	35.37	0.8943	35.29	0.8907	35.14	0.8816	35.61	0.8964	35.26	0.8856	35.49	0.8959	**36.56**	**0.9115**
Pond	30.70	0.8167	32.11	0.8542	32.08	0.8524	31.94	0.8342	32.21	0.8555	31.92	0.8348	32.18	0.8555	**33.89**	**0.8716**
Port	26.67	0.7986	28.50	0.8596	28.55	0.8679	28.31	0.8497	28.76	0.8635	28.33	0.8501	28.81	0.8638	**29.49**	**0.8789**
Railway Station	26.78	0.6793	28.72	0.7738	28.77	0.7786	28.55	0.7654	28.91	0.7789	28.51	0.7667	28.88	0.7780	**29.87**	**0.7979**
Resort	26.79	0.7029	28.52	0.7799	28.53	0.7801	27.31	0.7406	28.72	0.7846	27.36	0.7469	28.71	0.7849	**29.56**	**0.7936**
River	30.37	0.7402	31.55	0.7891	31.51	0.7876	31.04	0.7784	31.62	0.7906	31.11	0.7841	31.63	0.7909	**32.27**	**0.8016**
School	27.41	0.7237	29.36	0.8044	29.31	0.7997	29.06	0.7915	29.55	0.8089	29.09	0.7921	29.54	0.8104	**30.13**	**0.8269**
S-Residential	26.66	0.6006	27.71	0.6728	27.69	0.6736	27.56	0.6621	27.84	0.6759	27.59	0.6639	27.88	0.6759	**28.89**	**0.6956**
Square	28.55	0.7391	30.84	0.8200	30.87	0.8238	30.53	0.8006	31.03	0.8237	30.59	0.8027	31.00	0.8251	**31.73**	**0.8357**
Stadium	27.16	0.7547	29.63	0.8387	29.57	0.8346	29.41	0.8216	29.82	0.8425	29.57	0.8276	29.77	0.8422	**30.54**	**0.8526**
Storage Tanks	25.65	0.6793	27.44	0.7664	27.33	0.7643	27.17	0.7469	27.61	0.7705	27.21	0.7486	27.60	0.7698	**28.56**	**0.7813**
Viaduct	26.97	0.6755	28.99	0.7757	28.72	0.7689	28.67	0.7559	29.16	0.7805	28.71	0.7613	29.11	0.7794	**30.16**	**0.7889**
Average	28.86	0.7382	30.65	0.8086	30.43	0.7988	30.28	0.7802	30.82	0.8121	30.51	0.7942	30.81	0.8124	**31.13**	**0.8229**

**Table 3:** Quantitative Results on AID, DOTA V1.0, DIOR and NWPU-RESISC45 Test Sets. The best result is shown in **boldface**.

Methods	#Param.	FLOPs	AID		DOTA V1.0		DIOR		NWPU-RESISC45		Average	
			**PSNR**	**SSIM**	**PSNR**	**SSIM**	**PSNR**	**SSIM**	**PSNR**	**SSIM**	**PSNR**	**SSIM**
Bicubic	-	-	28.86	0.7382	31.16	0.7947	28.57	0.7432	26.20	0.6873	28.70	0.7587
EDSR	43.09 M	823.34 G	30.65	0.8086	33.64	0.8648	30.63	0.8116	28.91	0.7458	30.96	0.8077
VDSR	2.55 M	36.78 G	30.43	0.7988	33.54	0.8563	30.15	0.8064	28.65	0.7495	30.69	0.8025
RCAN	15.59 M	261.01 G	30.82	0.8121	33.86	0.8680	30.85	0.8159	28.99	0.7681	31.13	0.8160
SRFlow	16.78 M	321.4 G	30.28	0.7802	33.58	0.8561	30.39	0.8026	29.05	0.7687	30.82	0.8019
SRGAN	2.79 M	139.26 G	30.51	0.7942	33.47	0.8526	30.45	0.8049	28.97	0.7712	30.85	0.8057
HAT	40.32 M	672.15 G	30.81	0.8124	33.99	0.8684	30.87	0.8161	29.19	0.7835	31.22	0.8201
SwinIR	5.85 M	152.42 G	30.85	0.8137	33.93	0.8671	30.75	0.8133	29.12	0.7812	31.16	0.8188
ESRT	8.7 M	187.5 G	30.75	0.8089	33.91	0.8675	30.89	0.8173	29.11	0.7821	31.17	0.8189
ESTNet	3.28 M	89.32 G	30.89	0.8158	34.28	0.8697	31.27	0.8257	29.78	0.7943	31.55	0.8263
SMSR	1.2 M	87.5 G	30.95	0.8176	34.32	0.8703	31.39	0.8276	29.71	0.7936	31.59	0.8272
Ours_s	**4.16 M**	**8.73 G**	31.04	0.8195	35.52	0.8856	32.24	0.8358	29.87	0.8049	32.17	0.8364
Ours	16.01 M	114.49 G	**31.13**	**0.8229**	**35.69**	**0.8881**	**32.31**	**0.8374**	**29.95**	**0.8078**	**32.27**	**0.8391**

**Table 4:** We present the PSNR results under various noise conditions and Anisotropic Gaussian blurs. All models are evaluated on the DIOR dataset across 11 representative kernel widths and noise intensities ranging between 0 and 10. The best results are shown in **boldface**.

Method	Noise	Anisotropic Gaussian Blur Kernels											Average
		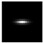	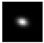	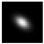	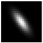	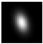	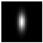	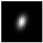	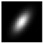	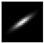	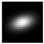	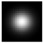	
Bicubic	0	27.59	27.36	26.62	26.12	26.01	26.53	26.75	26.03	26.31	25.95	25.76	26.37
EDSR		29.65	29.47	29.46	29.15	28.15	29.16	29.26	28.76	28.69	28.78	28.41	29.00
RCAN		29.86	29.81	29.15	27.48	27.91	27.17	29.03	27.74	27.65	27.96	29.15	28.44
HAT		29.87	29.66	29.25	28.61	28.37	28.75	29.17	28.65	28.79	28.55	28.18	28.89
VDSR		29.15	29.07	29.05	28.75	28.63	28.89	28.74	28.89	28.75	28.54	28.65	28.82
Ours_s		31.09	31.01	31.12	31.23	31.15	31.24	31.25	31.24	30.91	31.05	30.75	31.00
Ours		**31.25**	**31.27**	**31.36**	**31.45**	**31.37**	**31.49**	**31.35**	**31.35**	**31.14**	**31.29**	**30.95**	**31.21**
Bicubic	5	27.15	26.91	26.33	25.82	25.76	26.24	26.41	25.79	25.95	25.64	25.48	25.59
EDSR		28.42	28.48	28.25	27.39	27.64	27.97	28.23	27.31	27.67	27.19	27.14	27.52
RCAN		28.86	28.41	28.17	26.94	27.54	26.61	27.10	26.47	26.97	25.75	26.41	27.20
HAT		28.89	28.43	28.27	26.96	27.57	26.69	27.35	26.84	27.39	25.45	26.95	27.35
VDSR		27.97	27.95	27.84	26.69	27.15	26.59	27.58	26.03	27.15	27.29	26.88	26.74
Ours_s		29.97	29.94	29.01	28.65	29.13	28.89	29.12	28.56	29.63	29.21	29.12	29.20
Ours		**30.07**	**30.01**	**29.97**	**29.05**	**29.45**	**29.19**	**29.40**	**29.87**	**28.91**	**29.94**	**29.55**	**29.58**
Bicubic	10	26.68	26.34	25.85	25.31	25.19	25.74	25.91	25.26	25.59	25.16	24.91	24.72
EDSR		27.03	27.79	27.15	26.59	26.43	27.07	27.17	26.57	26.89	26.51	26.01	26.47
RCAN		27.23	27.07	27.18	26.89	26.69	27.28	27.54	26.83	26.24	26.72	26.19	26.53
HAT		27.76	27.16	27.57	26.93	26.81	27.54	27.73	26.93	26.46	26.78	26.27	26.81
VDSR		27.01	27.65	27.03	26.22	26.38	27.01	27.09	26.54	26.56	26.35	25.96	26.52
Ours_s		28.84	28.79	28.17	27.65	27.71	28.01	28.27	27.98	27.87	27.54	27.45	27.84
Ours		**29.05**	**28.99**	**28.87**	**28.01**	**27.98**	**28.15**	**28.57**	**28.10**	**28.05**	**27.99**	**27.64**	**28.31**

**Table 5:** Ablation results achieved by our method with different settings on AID.

Method	MPB	#Param.	FLOPs	PSNR	SSIM
Baseline	×	11.58 M	190.82 G	31.20	0.8257
Ours	✔	16.01 M	114.49 G	**31.13**	**0.8229**
Ours_s	✔	**4.16 M**	**8.73 G**	30.91	0.8174

## Data Availability

No new datasets were created or analyzed.

## References

[1] Shi W., Caballero J., Huszár F., Totz J., Aitken A.P., Bishop R., Rueckert D., Wang Z. Real-time single image and video super-resolution using an efficient sub-pixel convolutional neural network. Proceedings of the IEEE Conference on Computer Vision and Pattern Recognition.

[2] Zhang X., Zeng H., Guo S., Zhang L. (2022). Efficient long-range attention network for image super-resolution. The European Conference on Computer Vision.

[3] Liu X., Liu J., Tang J., Wu G. CATANet: Efficient Content-Aware Token Aggregation for Lightweight Image Super-Resolution. Proceedings of the Computer Vision and Pattern Recognition Conference.

[4] Peng G., Xie M., Fang L. (2023). Context-aware lightweight remote-sensing image super-resolution network. Front. Neurorobotics.

[5] Lin C., Mao X., Qiu C., Zou L. (2024). Dtcnet: Transformer-cnn distillation for super-resolution of remote sensing image. IEEE J. Sel. Top. Appl. Earth Obs. Remote Sens..

[6] Hou M., Huang Z., Yu Z., Yan Y., Zhao Y., Han X. (2024). CSwT-SR: Conv-swin transformer for blind remote sensing image super-resolution with amplitude-phase learning and structural detail alternating learning. IEEE Trans. Geosci. Remote Sens..

[7] Dong C., Loy C.C., He K., Tang X. (2015). Image super-resolution using deep convolutional networks. IEEE Trans. Pattern Anal. Mach. Intell..

[8] Kim J., Lee J.K., Lee K.M. Accurate image super-resolution using very deep convolutional networks. Proceedings of the IEEE Conference on Computer Vision and Pattern Recognition.

[9] Lim B., Son S., Kim H., Nah S., Mu Lee K. Enhanced deep residual networks for single image super-resolution. Proceedings of the IEEE Conference on Computer Vision and Pattern Recognition Workshops.

[10] Zhang Y., Li K., Li K., Wang L., Zhong B., Fu Y. Image super-resolution using very deep residual channel attention networks. Proceedings of the European Conference on Computer Vision (ECCV).

[11] Chen X., Wang X., Zhou J., Qiao Y., Dong C. Activating more pixels in image super-resolution transformer. Proceedings of the IEEE/CVF Conference on Computer Vision and Pattern Recognition.

[12] Kang X., Duan P., Li J., Li S. (2024). Efficient swin transformer for remote sensing image super-resolution. IEEE Trans. Image Process..

[13] Wang Y., Jin S., Yang Z., Guan H., Ren Y., Cheng K., Zhao X., Liu X., Chen M., Liu Y. (2024). TTSR: A transformer-based topography neural network for digital elevation model super-resolution. IEEE Trans. Geosci. Remote Sens..

[14] Liang J., Cao J., Sun G., Zhang K., Van Gool L., Timofte R. Swinir: Image restoration using swin transformer. Proceedings of the IEEE/CVF International Conference on Computer Vision.

[15] Wang L., Dong X., Wang Y., Ying X., Lin Z., An W., Guo Y. Exploring sparsity in image super-resolution for efficient inference. Proceedings of the IEEE/CVF Conference on Computer Vision and Pattern Recognition.

[16] Lu Z., Li J., Liu H., Huang C., Zhang L., Zeng T. Transformer for single image super-resolution. Proceedings of the IEEE/CVF Conference on Computer Vision and Pattern Recognition.

[17] Xia P., Peng L., Di X., Pei R., Wang Y., Cao Y., Zha Z.J. (2024). S3mamba: Arbitrary-scale super-resolution via scaleable state space model. arXiv.

[18] Di X., Peng L., Xia P., Li W., Pei R., Cao Y., Wang Y., Zha Z.J. Qmambabsr: Burst image super-resolution with query state space model. Proceedings of the Computer Vision and Pattern Recognition Conference.

[19] Liu J., Yuan Z., Pan Z., Fu Y., Liu L., Lu B. (2022). Diffusion model with detail complement for super-resolution of remote sensing. Remote Sens..

[20] Liebel L., Körner M. (2016). Single-image super resolution for multispectral remote sensing data using convolutional neural networks. Int. Arch. Photogramm. Remote Sens. Spat. Inf. Sci..

[21] Xu W., Guangluan X., Wang Y., Sun X., Lin D., Yirong W. High quality remote sensing image super-resolution using deep memory connected network. Proceedings of the IGARSS 2018-2018 IEEE International Geoscience and Remote Sensing Symposium.

[22] Ren C., He X., Qing L., Wu Y., Pu Y. (2021). Remote sensing image recovery via enhanced residual learning and dual-luminance scheme. Knowl.-Based Syst..

[23] Dong X., Sun X., Jia X., Xi Z., Gao L., Zhang B. (2020). Remote sensing image super-resolution using novel dense-sampling networks. IEEE Trans. Geosci. Remote Sens..

[24] Lei S., Shi Z., Mo W. (2021). Transformer-based multistage enhancement for remote sensing image super-resolution. IEEE Trans. Geosci. Remote Sens..

[25] Li Z., Li L., Liu B., Cao Y., Zhou W., Ni W., Yang Z. (2023). Spectral-learning-based transformer network for the spectral super-resolution of remote-sensing degraded images. IEEE Geosci. Remote Sens. Lett..

[26] Zhang M., Zhang C., Zhang Q., Guo J., Gao X., Zhang J. ESSAformer: Efficient transformer for hyperspectral image super-resolution. Proceedings of the IEEE/CVF International Conference on Computer Vision.

[27] Zhu Q., Zhang G., Zou X., Wang X., Huang J., Li X. (2024). Convmambasr: Leveraging state-space models and cnns in a dual-branch architecture for remote sensing imagery super-resolution. Remote Sens..

[28] Zhi R., Fan X., Shi J. (2024). MambaFormerSR: A lightweight model for remote-sensing image super-resolution. IEEE Geosci. Remote Sens. Lett..

[29] Li H., Yan C., Lin S., Zheng X., Zhang B., Yang F., Ji R. (2020). Pams: Quantized super-resolution via parameterized max scale. The European Conference on Computer Vision.

[30] Hong C., Kim H., Baik S., Oh J., Lee K.M. Daq: Channel-wise distribution-aware quantization for deep image super-resolution networks. Proceedings of the IEEE/CVF Winter Conference on Applications of Computer Vision.

[31] Hong C., Baik S., Kim H., Nah S., Lee K.M. (2022). Cadyq: Content-aware dynamic quantization for image super-resolution. The European Conference on Computer Vision.

[32] Tu Z., Hu J., Chen H., Wang Y. Toward accurate post-training quantization for image super resolution. Proceedings of the IEEE/CVF Conference on Computer Vision and Pattern Recognition.

[33] Jacob B., Kligys S., Chen B., Zhu M., Tang M., Howard A., Adam H., Kalenichenko D. Quantization and training of neural networks for efficient integer-arithmetic-only inference. Proceedings of the IEEE Conference on Computer Vision and Pattern Recognition.

[34] Lee N., Ajanthan T., Torr P.H. (2018). Snip: Single-shot network pruning based on connection sensitivity. arXiv.

[35] Tanaka H., Kunin D., Yamins D.L., Ganguli S. (2020). Pruning neural networks without any data by iteratively conserving synaptic flow. Adv. Neural Inf. Process. Syst..

[36] Wang C., Zhang G., Grosse R. (2020). Picking winning tickets before training by preserving gradient flow. arXiv.

[37] Mocanu D.C., Mocanu E., Stone P., Nguyen P.H., Gibescu M., Liotta A. (2018). Scalable training of artificial neural networks with adaptive sparse connectivity inspired by network science. Nat. Commun..

[38] Evci U., Gale T., Menick J., Castro P.S., Elsen E. Rigging the lottery: Making all tickets winners. Proceedings of the International Conference on Machine Learning, PMLR.

[39] Liu Z., Li J., Shen Z., Huang G., Yan S., Zhang C. Learning efficient convolutional networks through network slimming. Proceedings of the IEEE International Conference on Computer Vision.

[40] Gordon A., Eban E., Nachum O., Chen B., Wu H., Yang T.J., Choi E. Morphnet: Fast & simple resource-constrained structure learning of deep networks. Proceedings of the IEEE Conference on Computer Vision and Pattern Recognition.

[41] Frankle J., Carbin M. (2018). The lottery ticket hypothesis: Finding sparse, trainable neural networks. arXiv.

[42] Liu S., Chen T., Atashgahi Z., Chen X., Sokar G., Mocanu E., Pechenizkiy M., Wang Z., Mocanu D.C. (2021). Deep ensembling with no overhead for either training or testing: The all-round blessings of dynamic sparsity. arXiv.

[43] Willette J., Lee H., Hwang S.J. (2025). Delta Attention: Fast and Accurate Sparse Attention Inference by Delta Correction. arXiv.

[44] Zhang J., Xiang C., Huang H., Wei J., Xi H., Zhu J., Chen J. (2025). Spargeattn: Accurate sparse attention accelerating any model inference. arXiv.

[45] Gao Y., Guo S., Cao S., Xia Y., Cheng Y., Wang L., Ma L., Sun Y., Ye T., Dong L. (2025). SeerAttention-R: Sparse Attention Adaptation for Long Reasoning. arXiv.

[46] Acharya S., Jia F., Ginsburg B. (2024). Star attention: Efficient llm inference over long sequences. arXiv.

[47] Liu Z., Lin Y., Cao Y., Hu H., Wei Y., Zhang Z., Lin S., Guo B. Swin transformer: Hierarchical vision transformer using shifted windows. Proceedings of the IEEE/CVF International Conference on Computer Vision (ICCV).

[48] Xia G.S., Hu J., Hu F., Shi B., Bai X., Zhong Y., Zhang L., Lu X. (2017). AID: A benchmark data set for performance evaluation of aerial scene classification. IEEE Trans. Geosci. Remote Sens..

[49] Xia G.S., Bai X., Ding J., Zhu Z., Belongie S., Luo J., Datcu M., Pelillo M., Zhang L. DOTA: A large-scale dataset for object detection in aerial images. Proceedings of the IEEE Conference on Computer Vision and Pattern Recognition.

[50] Li K., Wan G., Cheng G., Meng L., Han J. (2020). Object detection in optical remote sensing images: A survey and a new benchmark. ISPRS J. Photogramm. Remote Sens..

[51] Cheng G., Han J., Lu X. (2017). Remote sensing image scene classification: Benchmark and state of the art. Proc. IEEE.

[52] Wang Z., Bovik A.C., Sheikh H.R., Simoncelli E.P. (2004). Image quality assessment: From error visibility to structural similarity. IEEE Trans. Image Process..

[53] Xiao Y., Yuan Q., Jiang K., He J., Wang Y., Zhang L. (2023). From degrade to upgrade: Learning a self-supervised degradation guided adaptive network for blind remote sensing image super-resolution. Inf. Fusion.

[54] Wang L., Guo Y., Dong X., Wang Y., Ying X., Lin Z., An W. (2023). Exploring fine-grained sparsity in convolutional neural networks for efficient inference. IEEE Trans. Pattern Anal. Mach. Intell..

[55] Zhan Z., Gong Y., Zhao P., Yuan G., Niu W., Wu Y., Zhang T., Jayaweera M., Kaeli D., Ren B. Achieving on-mobile real-time super-resolution with neural architecture and pruning search. Proceedings of the ICCV.

[56] Zhang Y., Zhang K., Van Gool L., Danelljan M., Yu F. Lightweight image super-resolution via flexible meta pruning. Proceedings of the ICML.

[57] Wang L., Dong X., Wang Y., Liu L., An W., Guo Y. Learnable lookup table for neural network quantization. Proceedings of the CVPR.

[58] Yamamoto K. Learnable companding quantization for accurate low-bit neural networks. Proceedings of the CVPR.

[59] Zhang X., Zhang Y., Yu F. HiT-SR: Hierarchical transformer for efficient image super-resolution. Proceedings of the ECCV.

[60] Zamfir E., Wu Z., Mehta N., Zhang Y., Timofte R. See more details: Efficient image super-resolution by experts mining. Proceedings of the ICML.

